# Ultrasound Guidance for Deep Peripheral Nerve Blocks: A Brief Review

**DOI:** 10.1155/2011/262070

**Published:** 2011-07-27

**Authors:** Anupama Wadhwa, Sunitha Kanchi Kandadai, Sujittra Tongpresert, Detlef Obal, Ralf Erich Gebhard

**Affiliations:** ^1^Department of Anesthesiology and Perioperative Medicine, University of Louisville Hospital, University of Louisville, 530 S. Jackson Street, Louisville, KY 40202, USA; ^2^Outcomes Research Consortium, Cleveland Clinic, Cleveland, OH 44195, USA; ^3^Department of Anesthesiology, Miller School of Medicine, University of Miami, Miami, FL 33136, USA

## Abstract

Nerve stimulation and ultrasound have been introduced to the practice of regional anesthesia mostly in the last two decades. Ultrasound did not gain as much popularity as the nerve stimulation until a decade ago because of the simplicity, accuracy and portability of the nerve stimulator. Ultrasound is now available in most academic centers practicing regional anesthesia and is a popular tool amongst trainees for performance of nerve blocks. This review article specifically discusses the role of ultrasonography for deeply situated nerves or plexuses such as the infraclavicular block for the upper extremity and lumbar plexus and sciatic nerve blocks for the lower extremity. Transitioning from nerve stimulation to ultrasound-guided blocks alone or in combination is beneficial in certain scenarios. However, not every patient undergoing regional anesthesia technique benefits from the use of ultrasound, especially when circumstances resulting in difficult visualization such as deep nerve blocks and/or block performed by inexperienced ultrasonographers. The use of ultrasound does not replace experience and knowledge of relevant anatomy, especially for visualization of deep structures. In certain scenarios, ultrasound may not offer additional value and substantial amount of time may be spent trying to find relevant structures or even provide a false sense of security, especially to an inexperienced operator. We look at available literature on the role of ultrasound for the performance of deep peripheral nerve blocks and its benefits.

## 1. Introduction

For decades, regional anesthesia has been performed mainly with the help of nerve stimulation [1]. Transitioning from nerve stimulation to ultrasound-guided blocks alone or in combination is beneficial in certain types of regional blocks and scenarios. However, not every patient undergoing a regional anesthesia technique benefits from the use of ultrasound, especially under circumstances resulting in difficult visualization and/or block performed by inexperienced ultrasonographers. The aim of this paper is to specifically discuss the role of ultrasonography for deeply situated nerves or plexuses such as the infraclavicular block for the upper extremity and lumbar plexus and sciatic nerve blocks for the lower extremity. While the authors realize that very few randomized controlled trials (RCTs) have been performed comparing the use of ultrasound with nerve stimulation for performing blocks in deeply located nerves or plexuses, we will attempt to draw conclusions from the existing RCTs.

## 2. History of Ultrasound Use

About 22 years ago, Ting and Sivagnanaratnam [2] were among the first to utilize ultrasonography to confirm the location of the needle used to perform blocks and observe the spread of local anesthetic while performing axillary nerve blocks. They reported 100% success rate of axillary nerve blocks with no complications during this very first study, and that they were able to visualize the needle tip and axillary anatomy at all times. Subsequently, almost five years later Kapral et al. [3] demonstrated that use of ultrasound for supraclavicular blocks resulted in safe and more effective anesthesia than axillary blocks for the brachial plexus distribution [3]. The same group from Vienna later demonstrated improved success of “three-in-one” lower extremity blocks performed under ultrasound guidance compared with nerve stimulation [4]. They further showed that local anesthetic requirements to produce an effective block were reduced with ultrasound guidance [5]. The use of ultrasound localization of nerves was further advanced when researchers in Toronto demonstrated high quality images of the brachial plexus with ultrasound [6]. They also confirmed the findings by Urmey [7] that contact of a stimulating needle with a nerve does not necessarily elicit a motor response when utilizing nerve stimulation [6, 8].

## 3. Advantages of Ultrasound Use

Ultrasound guidance makes sense and is intuitive to use for superficial blocks such as supraclavicular, interscalene, axillary, and femoral blocks. These areas are easily visualized with the use of high frequency, linear array transducers that provide very high-resolution images of the brachial plexus. Some of upper extremity blocks like the supraclavicular approach to the brachial plexus have even regained popularity because of the use of ultrasonography in this area. While this has not been proven in randomized controlled trials, visualization of the subclavian artery and the pleura may reduce the incidence of accidental puncture of these structures and consequently reduce the incidence of hematoma and/or pneumothorax. In addition, avoiding blood vessels in general should minimize the chance of local anesthetic toxicity by avoidance of direct injection into the blood stream. Ease of visualization does result in increased success rate in experienced hands and reduced performance time when compared with nerve stimulation alone. Another definite and unique advantage demonstrated with the use of ultrasound guidance is the reduced amount of local anesthetic required to block a variety of nerves and plexuses [5, 9–11]. This is due to the ability to visualize the spread of local anesthetic surrounding a nerve or a plexus, decreasing the need of large amounts of local anesthetic. Although ultrasound has been proven an invaluable tool for regional anesthesia, its use for deep nerve blocks may prove to be more difficult than their superficial counterparts [12, 13]. This is supported by the very few ultrasound studies available for these blocks.

## 4. Evidence of Use of Ultrasonography for Deep Nerve Blocks

We performed a literature search using Pubmed with each of the three nerve blocks as keywords. We selected published RCTs comparing ultrasound with neurostimulation, for each of the deep peripheral nerve blocks like the sciatic, lumbar plexus, and infraclavicular blocks. Studies were selected from 1993 till present (Table 1). All case series, case reports, and nonrandomized studies were excluded. 

### 4.1. Sciatic Nerve at and above the Subgluteal Level

Nerves can generally be identified by their relationship with other bony landmarks or major blood vessels. The sciatic nerve is recognized by its location between two bony structures: the ischial tuberosity and the greater trochanter. In the gluteal and subgluteal area, the bulk of the gluteus maximus muscle and the adipose tissue make it difficult to identify any deep “soft” structures. In the morbidly obese patients, it may be difficult to recognize even the bony structures. Previous investigators, who have attempted to identify easy and reliable internal ultrasound landmarks for the localization of the sciatic nerve, suggested that locations as much as 7–10 cm distal to the subgluteal fold may be advantageous for sciatic nerve visualization with ultrasound [12, 14]. Negotiating the sciatic nerve at a level above the subgluteal fold may be difficult with the use of just ultrasound in certain patient populations such as the morbidly obese or patients with positioning issues. Popliteal approach is common for sciatic nerve blocks; this may explain the paucity of studies for ultrasound-guided sciatic nerve block at higher levels. We were able to identify only one study comparing nerve stimulation and ultrasound guidance at the subgluteal level [15]. Using the “up and down” technique, the main outcome parameter was minimal local anesthetic volume necessary to achieve a complete sensory and motor block. Subjects in the ultrasound-guided group were reported to have a 37% reduction in mepivacaine requirements compared with those in the nerve stimulation-guided group. We speculate that the reason for the lack of studies regarding this anatomical approach may be related to the sheer bulk of the gluteus maximus muscle, making sciatic nerve visualization difficult with an ultrasound machine, while nerve stimulation using solid anatomical landmarks reliably provides quick and easy access to the sciatic nerve. Abbas and Brull [16] mentioned their routine use of ultrasound at this level in a letter to the editor; however, it remains to be seen if addition of ultrasound to nerve stimulation offers any advantage to this block. 

Given that only one study has been performed comparing the subgluteal sciatic nerve block with the two guidance techniques, it would be reasonable only to comment on the reduced local anesthetic requirement with ultrasound.

### 4.2. Lumbar Plexus Block

Lumbar plexus blocks are considered advanced blocks by regional anesthesiologists because of the level of difficulty of the block and the potential complications associated with them. Consequently, several case reports of retroperitoneal hematoma and bleeding [17–19], renal subcapsular hematoma [20], and epidural and contralateral spread of local anesthetic have been published [21, 22].

In one of the very first studies, Kirchmair et al. [23] looked at the paravertebral anatomy for ultrasound guided posterior lumbar plexus block. They identified the psoas, quadratus lumborum, and the erector spinae muscles along with the transverse processes at L2–L5 levels. They were able to identify the sono-anatomy in 100% of all volunteers with normal BMI; however, sonography was unfeasible in 20% of patients who were overweight and in 33% of obese volunteers. Thus, ultrasonography may provide no additional benefit over landmarks in overweight patients who may have difficult-to-feel anatomical landmarks. Their subsequent study on cadavers [24] demonstrated good accuracy of ultrasound in reaching the psoas muscle; conversely this study does not give any information about the success rate or efficacy of ultrasound in improving success. 

To date, only a single RCT [25] has been reported comparing use of ultrasound versus nerve stimulation guidance for performance of lumbar plexus blocks in patients undergoing total hip replacement. In the ultrasound group, 22% of patients (*n* = 23) and 30% of patients in the nerve stimulation group (*n* = 23) showed incomplete sensory and/or motor block in one of clinically relevant territories of distribution of the lumbar plexus (L2–L4) at 30 minutes after injection of local anesthetic. These patients required an additional bolus of local anesthetic via the lumbar plexus catheter before incision (*P* = 0.36). The average time to readiness for surgery, as defined in the study protocol, was about 7 minutes faster in the ultrasound group (*P* = 0.04). A similar number of patients in both groups required general anesthesia to complete surgery as a result of discomfort referred to pain in the L2-L3 lumbar plexus dermatomes (*P* = 0.73). There were no statistical differences in postoperative pain scores between the two groups at 12 hours (NRS rest = 3 ± 2 in ultrasound group and 3 ± 3 in the nerve stimulation group) and 24 hours (NRS rest = 3 ± 2 in ultrasound group and 2 ± 2 in nerve stimulation group). Average local anesthetic consumption in the first 24 h was also similar between the groups. Thus in patients undergoing primary total hip replacement, ultrasound assistance with nerve stimulation guidance to the lumbar plexus allowed a faster readiness for surgery while often requiring a lower local anesthetic volume than the nerve stimulation technique alone. This may be a result of the reduction of needle redirections that practitioners have to perform to localize the lumbar plexus. The lumbar plexus is located at a depth of 7-8 cm and even deeper in patients with high BMI, and consequently not easily identifiable with ultrasound. High-definition ultrasonography may offer potential advantages in the administration of peripheral nerve blockade. Ilfield et al. [26] provide evidence that prepuncture ultrasound accurately predicts maximal transverse process depth to within 1 cm of its actual location. In addition, the cephalad-caudad location of each transverse process can be estimated with ultrasound to help guide the needle introduction site to either intersect or avoid needle-process contact, whichever the practitioner prefers. Nevertheless, the two techniques seem to be comparable in terms of postoperative local anesthetic consumption and pain scores [25]. Neither of the two techniques seems to offer a clear advantage over the other one. Therefore, we may conclude from the limited data that using a combined prepuncture, ultrasound imaging and neuro-stimulation are likely to reduce the number of needle passes and may position the needle tip closer to the plexus.

Specific complications like retroperitoneal hematoma associated with the lumbar plexus block occur very rarely. Most of them may be explained by either patients being anticoagulated [27] or difficulty during block performance leading to multiple block attempts or by inexperience of the operator [20].

Case reports [13, 28] have attempted to describe the sono-anatomy of location of lumbar plexus between the lumbar transverse processes and the psoas muscle. Some authors have described visualizing the plexus with ultrasound, while others were unable to visualize the plexus itself; but they described the relevant anatomy and the ability to identify the lower pole of kidney [23, 24].

### 4.3. Infraclavicular Block

Infraclavicular region is particularly suitable for placement of catheters because of the ability to insert and stabilize the catheter in this area. Compared with the supraclavicular area, where the trunks of the brachial plexus are in close proximity to each other, the brachial plexus divides into divisions/cords below the clavicle. These surround the axillary artery laterally, medially, and posteriorly. Thus, methods that use multiple injection techniques for this block are likely to achieve higher success rates than single injection techniques. Use of hand held Doppler has shown to improve success rate even with a single injection of local anesthetic [29]. We reviewed the available literature to evaluate the usefulness of ultrasound over nerve stimulation in performing these blocks. 

In one of the first studies, Wu and colleagues [35] report that ultrasound imaging aided the performance of infraclavicular block and easily blocked the ulnar segment of the medial cord and the intercostobrachial nerves, resulting in enhanced tourniquet pain prevention. However, they mentioned that the distance to the plexus was deeper with this approach, requiring user experience and that the anesthesiologists needed to utilize delicate manipulation when using the anatomical landmark technique. While complete blocks in this investigation were achieved with ultrasound guidance in eight out of nine patients, subclavian artery puncture still occurred on three occasions. 

Ootaki et al. [36] used ultrasonography for infraclavicular block in a case series of 60 patients; surgery was successfully performed without supplementation of any other anesthetics or analgesics in 95% of cases. Complete sensory block was obtained in 100% for the musculocutaneous and medial antebrachial cutaneous nerves, 96.7% for the median nerve, and 95% for the ulnar and radial nerves. A complete motor block was achieved in 100% for the musculocutaneous nerve, 96.7% for the median nerve, 90% for the ulnar nerve, and 93.3% for the radial nerve. No complications were identified.

Five randomized controlled studies have compared ultrasound-guided and nerve stimulator-guided infraclavicular blocks in adult patients [32–34, 37]. All studies reported a high success rate with either ultrasound- or with nerve stimulation-guidance, without being able to demonstrate a significant difference between the two modes of nerve identification [32–34, 38]. However, visualization of major anatomic structures by ultrasound appeared to shorten the time to achieve a successful block [33, 34]. While most studies failed to demonstrate a better quality of nerve blockade with one method over the other [38], there was a trend toward a higher success rate in the ultrasound-guided groups [32]. Although limited by sample size, Gurkan demonstrated that the complication rate (e.g., vascular puncture) was lower in the ultrasound-guided than in the nerve stimulator-guided group [38].

In contrast to the previous studies, Dingemans et al. [30] compared the combination of nerve stimulation and ultrasound guidance with ultrasound guidance alone for infraclavicular blocks with residents executing the procedures in all patients. In the “ultrasound only group”, a minimum number of injections necessary to visualize local anesthetic spread posterior to and on each side of the axillary artery was performed. The “combined group” received a single injection of local anesthetic after obtaining a distal response to nerve stimulation. They needed one injection for a U-shaped spread around the axillary artery in 76% of patients, two injections in 16% of patients, and three injections in 8% of patients. Dingeman et al. found that infraclavicular nerve blocks were performed faster and with a higher success rate in obtaining a complete block in the ultrasound only group. 

In terms of complication avoidance, some case reports have demonstrated that the pressure applied with the ultrasound probe in the infraclavicular area can collapse blood vessels and lead to a negative aspiration test, even when needle position is actually intravascular [39]. 

## 5. Conclusions

Although existing studies suggest that the time required to perform peripheral nerve blocks is shortened with the use of ultrasound, the time required to perform an initial ultrasound exam is not included in the total time reported in any of these investigations. Given the limited number of lower extremity studies, it is difficult to comment on the specific advantages of ultrasound with the exemption of a reduced local anesthetic volume required for lumbar plexus blocks. Routine use of ultrasound without the use of a nerve stimulator for deep nerve blocks may give a false sense of security regarding avoidance of complications, as the tip of needle may not be visualized at all times or not at all because of the depth of the neural structures. Consequently, cases of recognized or unrecognized arterial punctures have been reported even with the use of an ultrasound. 

Ultrasound offers obvious advantages, but the operator needs to recognize the potential for pitfalls during clinical use related to difficulties with needle visualization and incorrect identification of structures. Even small involuntary movements may lead to insertion into a vascular structure and subsequent local anesthetic toxicity. Visualization of deeper structures may require more pressure to be applied to the probe in order to visualize the target nerve, causing the collapse of blood vessels leading to a false-negative aspiration test. 

In summary, without discounting the advantages of adding ultrasound guidance to the armamentarium of regional anesthesia, it would be fair to state that based on the current literature, ultrasound does not replace experience and knowledge of relevant anatomy, especially for visualization of deep structures. In certain scenarios, ultrasound may not offer additional value and substantial amount of time may be spent trying to find relevant structures. In other cases, it may provide a false sense of security, especially to an inexperienced operator. More studies are needed to define the role of ultrasound for the performance of deep peripheral nerve blocks and validate its benefits.

## Figures and Tables

**Table 1:** 

Study	No. patients	Onset of block time (min)	Time for procedure completion (min)	Success rate (%)	Time to resolution of motor block (min)	Local anesthetic volume	Complications
Dingemans et al. [30]	72	NA	3.1 (US) versus 5.2 (USPNS)	92% (US) versus 74% (USPNS)	NA	Lidocaine 1.5% and Bupivacaine.125% with epi 0.5 mL/kg	NA

Dhir and Ganapathy [31]	66	28 (NS) versus 24 (SC) versus 21(USPNS)	6 (NS) versus 8 (SC) versus 6 (USPNS)	59% (NS) versus 58% (SC) versus 96% (USPNS)	266 (NS) versus 247 (SC) versus 246 (USPNS)	30 mL of Ropivacaine 5 mg/mL with epi 2.5 *μ*g/mL	Secondary catheter failure 9% (US) versus 86% (USPNS)

Sauter et al. [32]	80	13.9 (US) versus 13.7 (USPNS)	4.1 (US) versus 4.3 (USPNS)	95% (US) versus 85% (USPNS)	NA	20 mL Lidocaine 0.5% & 20 mL Bupivacaine, 20 mL Levo-Bupivacaine 0.5% with epi 5 mg/mL	Vascular puncture 6.6%

Brull et al. [33]	103	5 (US) versus 10.5 (USPNS)	5 (US) versus 10.5 (USPNS)	85% (US) versus 65% (USPNS)	NA	Lidocaine 2% 15 mL and 15 mL Bupivacaine 0.5% with epi	No difference in complications in the two groups

Taboada et al. [34]	70	17 (US) versus 19 (USPNS)	3 (US) versus 6 (USPNS)	89% (US) versus 91% (USPNS)	237 (US) versus 247 (USPNS)	NA	NA

US: ultrasound, USPNS: ultrasound plus peripheral nerve stimulation, SC: stimulating catheter.
